# A Genetic Screen in *Drosophila* To Identify Novel Regulation of Cell Growth by Phosphoinositide Signaling

**DOI:** 10.1534/g3.119.400851

**Published:** 2019-11-08

**Authors:** Vishnu Janardan, Sanjeev Sharma, Urbashi Basu, Padinjat Raghu

**Affiliations:** National Centre for Biological Sciences-TIFR, GKVK Campus, Bellary Road, Bangalore, 560065, India

**Keywords:** Phosphoinositides, cell growth, metazoan, *Drosophila melanogaster*

## Abstract

Phosphoinositides are lipid signaling molecules that regulate several conserved sub-cellular processes in eukaryotes, including cell growth. Phosphoinositides are generated by the enzymatic activity of highly specific lipid kinases and phosphatases. For example, the lipid PIP_3_, the Class I PI3 kinase that generates it and the phosphatase PTEN that metabolizes it are all established regulators of growth control in metazoans. To identify additional functions for phosphoinositides in growth control, we performed a genetic screen to identify proteins which when depleted result in altered tissue growth. By using RNA-interference mediated depletion coupled with mosaic analysis in developing eyes, we identified and classified additional candidates in the developing *Drosophila melanogaster* eye that regulate growth either cell autonomously or via cell-cell interactions. We report three genes: *Pi3K68D*, *Vps34* and *fwd* that are important for growth regulation and suggest that these are likely to act via cell-cell interactions in the developing eye. Our findings define new avenues for the understanding of growth regulation in metazoan tissue development by phosphoinositide metabolizing proteins.

During metazoan development, tissue growth is underpinned by processes regulating cellular growth through molecular mechanisms leading to an accumulation of biomass, cell division or cell death. Several conserved signaling pathways such as the Insulin/Insulin-like growth factor signaling (IIS), mechanistic Target of Rapamycin (mTOR), Hedgehog, Wingless/Wnt, Notch and Hippo signaling are involved in this process. They control protein synthesis, initiation and progression of the cell cycle and apoptosis in the context of environmental factors that act as developmental cues. Inputs to these signaling systems include nutrients, systemically circulating hormones and even patterning or mechanical cues arising within individual tissues (Neto-Silva *et al.* 2009; Hariharan 2015).

Phosphoinositides are a family of phospholipids derived by the phosphorylation of phosphatidylinositol (PI). They form a physiologically important group of lipid messengers regulating cellular processes ranging from signaling, vesicular transport and cytoskeletal organization to transcription, RNA maturation, autophagy and cell survival (Balakrishnan *et al.* 2015; Fiume *et al.* 2015). The mono- [PI3P, PI4P and PI5P], bis- [PI(3,4)P_2_, PI(3,5)P_2_, PI(4,5)P_2_] and tris- [PI(3,4,5)P_3_] phosphorylated derivatives of PI are formed by the action of a set of kinases and phosphatases that control the highly selective phosphorylation of PI at positions three, four and five of the inositol ring. The synthesis and availability of PI itself is controlled by additional enzymes and transfer proteins including diacyl glycerol kinase (DGK), PI synthase (PIS), cytidine diphosphate diacylglycerol synthase (CDS) and PI transfer proteins (PITPs). The degradation of PI(4,5)P_2_ in the context of receptor activation is also mediated by phosphoinositide specific phospholipase C (PLC) enzymes. Together, this set of enzymes constitutes the control mechanism determining the cellular profile of phosphoinositides at any given time (Figure 1). Within cells, these reactions are organized such that compartment-specific profiles of phosphoinositides are present in eukaryotic cells (Fiume *et al.* 2015). In turn, the phosphoinositides themselves bind to and regulate the activity of a large number of effector proteins. This combination of enzymes and effector proteins constitute the phosphoinositide toolkit (Balakrishnan *et al.* 2015).

**Figure 1:**
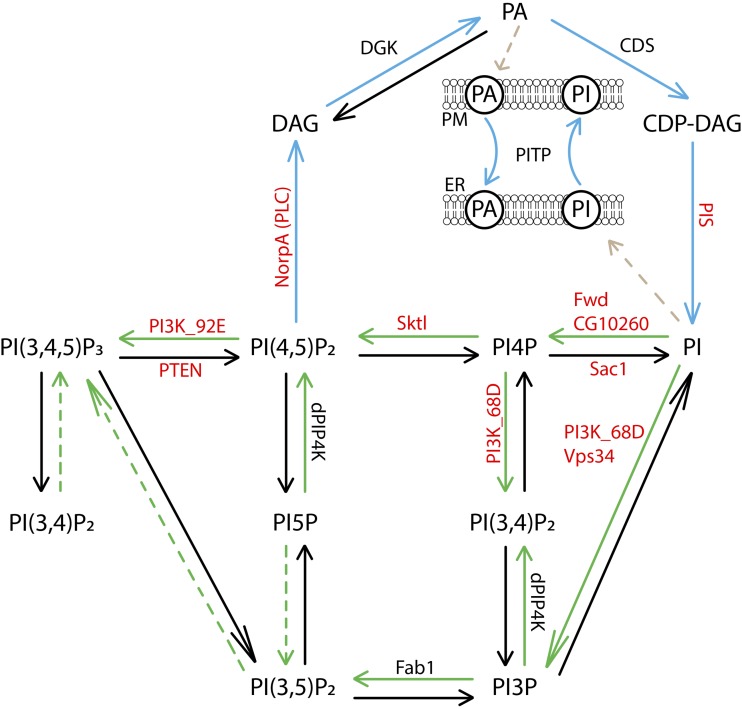
Phosphoinositide metabolism in eukaryotic cells. Phosphatidylinositol (PI) is synthesized from phosphatidic acid (PA) via cytidine diphosphate diacylglycerol (CDP-DAG). This involves the action of CDP-DAG synthase (CDS) and PI synthase (PIS) enzymes. Phosphatidylinositol thus formed can be phosphorylated to form phosphoinositides which are interconverted by various kinase and phosphatase reactions (green and black arrows respectively). Kinase reactions that are not well established are indicated by green dotted arrows. The phosphoinositide PI(4,5)P_2_ is converted to diacylglycerol (DAG) by the action of phospholipase C (*norpA*). DAG is then converted to PA and recycled back to form PI (blue arrows). The inset represents PI-transfer proteins (PITPs) that function to transfer PI (and PA) between membranes. All the genes identified to modulate growth in our screen are marked in red.

*Drosophila* has proved to be a powerful model system to study the physiological roles of genes involved in phosphoinositide metabolism, especially in the context of growth and development. Several studies conducted across a variety of tissues have implicated many phosphoinositide kinases and phosphatases in cellular processes such as the establishment of polarity, response to morphogens and growth factors, endocytosis and autophagy (Hassan *et al.* 1998; MacDougall *et al.* 2004; Yavari *et al.* 2010; Yan *et al.* 2011; Rousseau *et al.* 2013; Jiang *et al.* 2016). However, a systematic study comparing the roles of all known mediators of phosphoinositide metabolism in a single tissue is lacking. Mosaic screens using the *Drosophila* eye have been previously used to identify a number of genes that regulate cell growth, patterning and cell-cell interactions. Such assays allow side-by-side comparison of transgenic/mutant cells with wild-type cells. In this report, using a recently established CoinFLP system of generating eye mosaics (Bosch *et al.* 2015), we describe a targeted-RNAi screen that explores the role of almost all known *Drosophila* orthologs of phosphoinositide-metabolizing enzymes in regulating cell growth within the developing eye. We identify novel links between phosphoinositide metabolism and growth regulation and discuss plausible mechanisms through which these genes may modulate cell growth in a developing metazoan tissue.

## Materials and methods

### Fly culture and lines used in this study

Flies (*Drosophila melanogaster*) were reared on media containing cornmeal, dextrose, yeast powder, and agar along with antibacterial and antifungal agents. Flies were maintained at 25° and 50% relative humidity.

*Red Oregon-R (ROR)* flies were used as the wild-type strain. The other lines used were (a) *eyFLPase*, *UAS-dcr2*; *Sp/CyO*; *UAS-white^RNAi^* (b) *CoinFLP-Gal4*, *UAS-GFP* (II) and (c) *Act>y^+^>Gal4*, *UAS-GFP* (Kind gift from Dr. Iswar Hariharan, UC, Berkeley). Sources and stock numbers of the various RNAi lines used are listed in Table 1.

**Table 1:** List of *Drosophila* phosphoinositide signaling genes used for the screen and a summary of the results. Gene names along with their FBgn numbers, CG numbers and closest human orthologs are listed. For each of these genes, the various VDRC and TRiP RNAi lines used and the phenotypes observed with each are reported. In the CoinFLP mosaic analysis, the severity of the phenotype is represented by ‘+’, with ‘+++’ being the most severe (wherein the knockdown clones are completely absent) and ‘+’ being the mildest. The positive hits are represented by bold text

						CoinFLP RNAi screen				Act > y+>Gal4
FBgn	CG #	*Gene name/ Symbol (as in FlyBase)*	*Closest human orthologs*	VDRC #	BL #	Wild-type	Cell elimination	Overgrowth	Others	
FBgn0037339	CG2929	Pi4KIIα	PI4K2A	v25458			+			
				v25459			++			
				v40995		+				
				v110687		+				
**FBgn0267350**	**CG10260**	**PI4KIIIα**	PI4KA	v105614			+++			
				v15993			+++			Lethal
					BL35643		+			
					BL35256		+			
**FBgn0004373**	**CG7004**	**four wheel drive (fwd)**	PI4KB	v110159			++			
				v27786			+++		Smaller eyes. May affect patterning	Normal eyes
				v27785		+				
					BL29396		++			
					BL31187		+			
					BL35257		+			
**FBgn0016984**	**CG9985**	**skittles**		v6231			+++		Smaller eyes. May affect patterning	Smaller eyes
				v6229			+++			
					BL35198		+			
					BL27715		+++		Crunched eyes. May affect patterning	
FBgn0034789	CG3682	PIP5K59B	PIP5K1A	v108104		+				
				v47027		+				
				v47029		+				
FBgn0039924	CG17471	PIP4K	PIP4K2B		BL35338	+				
					BL35660	+				
					BL65891	+				
FBgn0028741	CG6355	fab1	PIKFYVE	v27591			+			
				v27592		+				
					BL35793	+				
**FBgn0015279**	**CG4141**	**Pi3K92E (Dp110)**	PIK3CD	v107390			+			
				v38986		+				
				v38985			+++		Smaller eyes	Smaller eyes
					BL35798		+			
					BL27690		+++		Smaller eyes	
					BL61182		++			
**FBgn0015278**	**CG11621**	**Pi3K68D**	PIK3C2A	v109582			++		Crunched eyes	
				v16240		+				
				v16239		+				
					BL35265	+				
					BL34621	+				
					BL31252		++			Normal eyes
**FBgn0015277**	**CG5373**	**Pi3K59F (Vps34)**	PIK3C3	v100296			+++		Crunched eyes and antennae	Normal eyes
					BL36056		+			
					BL33384		+			
					BL64011		++		Rough/ glossy clones	
**FBgn0020622**	**CG2699**	**Pi3K21B (Dp60)**	PIK3R3	v104179			+++		Crunched eyes. May affect patterning	Smaller eyes
				v33556			++			
					BL36810		+			
					BL38991		+++			
FBgn0025742	CG9115	myotubularin (mtm)	MTMR2	v29032		+				
					BL38339	+				
					BL31552		+			
					BL57298	+				
FBgn0030735	CG3632		MTMR4	v110167			+			
				v26254		+				
					BL38341	+				
FBgn0028497	CG3530		MTMR7/8	v110786		+				
				v26216			++			
				v26217			+++			
					BL38340	+				
					BL25864	+				
FBgn0035945	CG5026		MTMR9	v105674		+				
				v34915		+				
				v34916		+				
					BL42759	+				
					BL38309	+				
					BL57020	+				
**FBgn0026379**	**CG5671**	**Pten**	PTEN	v101475				+		
				v35371		+				
					BL25841			+		
					BL25967			+		
					BL33643			+		Larger eyes
FBgn0036058	CG6707		PIP4P1	v110291			+++		Smaller eyes	
				v44557			+			
				v44556		+				
					BL28316	+				
FBgn0259166	CG42271/ CG33248		INPP4A	v100176			++			
				v41672		+				
					BL29411	+				
**FBgn0283500**	**CG9128**	**Sac1**	SACM1L	v44376			+++			
				v37217			+++			
				v37216		+				
					BL56013		+++			Lethal
FBgn0031611	CG17840	FIG4	FIG4	v107084		+				
				v45037			+++			
				v45038			+++			
					BL38291	+				
					BL58063	+				
FBgn0023508	CG3573	Ocrl	INPP5B	v34649		+				
				v110796		+				
					BL34722	+				
FBgn0034691	CG6562	Synaptojanin (Synj)	SYNJ1	v46070		+				
					BL44420	+				
					BL34378	+				
					BL27489	+				
FBgn0030761	CG9784	Phosphoinositide 5-phosphatase		v108075		+				
				v30098			+			
					BL34723	+				
FBgn0036273	CG10426	INPP5E	INPP5E	v16048		+				
					BL41701	+				
					BL34037		+++			
FBgn0038890	CG7956		INPP5F	v22638		+				
				v22637					Smaller eyes. Rough/ glossy clones	
**FBgn0030670**	**CG9245**	**Phosphatidylinositol synthase (Pis)**	CDIPT (PIS)	v11852			+++			Lethal
				v106842			++			
					BL29383		+++			
					BL55602		+			
FBgn0004611	CG4574	Plc21C	PLCB1	v108395					Lethal	
				v26558		+				
				v26557		+				
					BL33719	+				
					BL32438	+				
					BL31269	+				
					BL31270	+				
FBgn0262738	CG3620	norpA (PLCβ)	PLCB4	v21490			+++			Smaller eyes
				v105676			++		May affect patterning	
					BL31113	+				
					BL31197		+			
FBgn0003218	CG11111	rdgB	PITPNM2	v6226					Rough/ glossy clones	
					BL28796	+				
FBgn0027872	CG17818	rdgBβ	PITPNC1	v19089		+				
				v104799		+				
					BL44523	+				
FBgn0003416	CG4200	small wing (PLCγ)	PLCG1	v7173			++			
				v7174			++			
				v108593		+				
					BL32385		+			
					BL32906	+				
					BL35604	+				
FBgn0010350	CG7962	Cds	CDS1/2		BL28075	+				
					BL58118	+				

### CoinFLP screen

**Figure 2:**
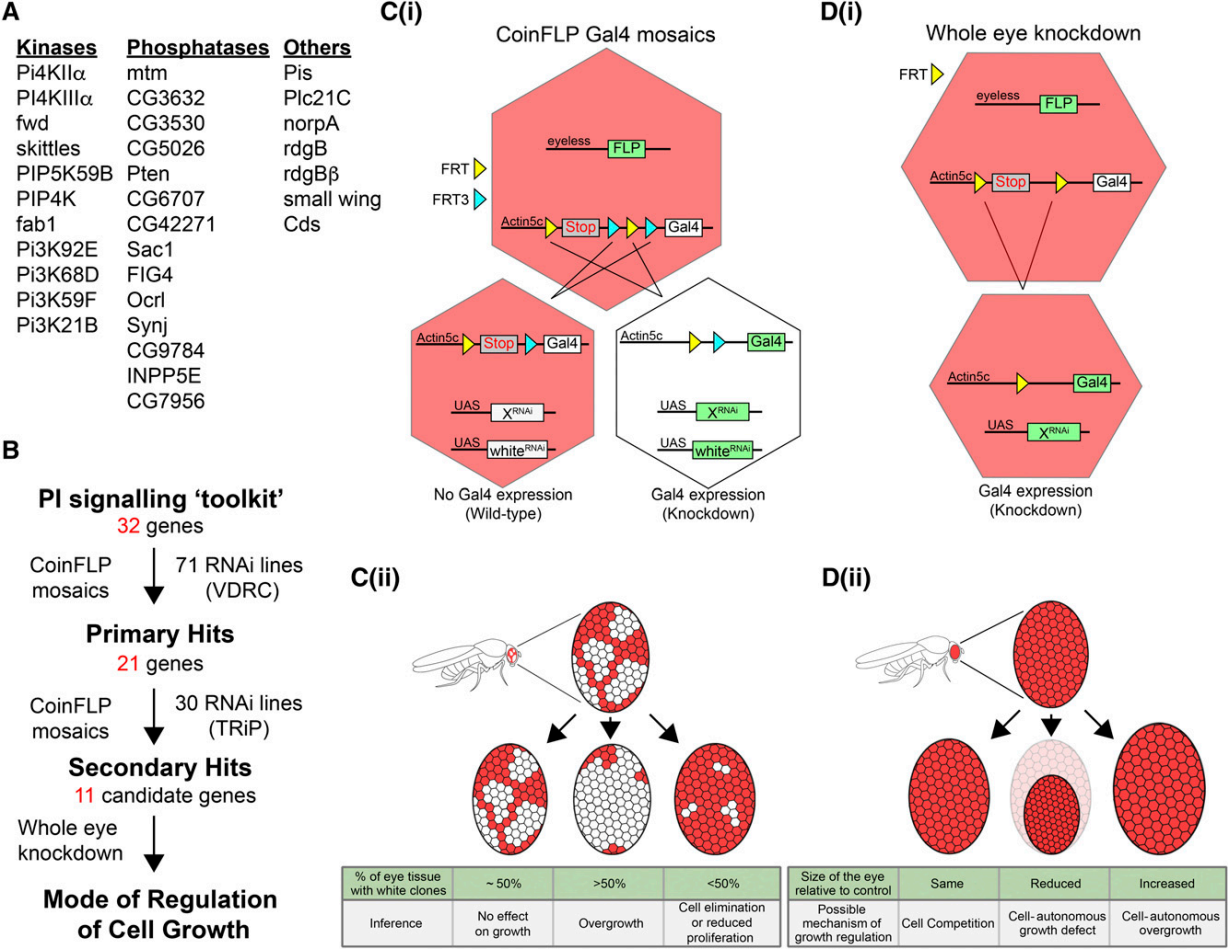
Overall strategy of the screen. (A) A list of all the genes screened. (B) A flowchart of the screen indicating number of genes screened and number of RNAi lines selected at each stage. (C) Graphical representation of (i) the CoinFLP system that results in two populations of cells. One population undergoes FLP mediated recombination at the FRT3 sites (cyan triangles), thus retaining the STOP cassette (gray) and not expressing Gal4. Ommatidia formed by these cells appear red in color. In the other population, recombination between FRT sites (yellow triangles) results in removal of the STOP cassette, thus activating Gal4 under the *Actin 5c* promoter. Ommatidia formed by these cells appear white in color due to expression of white^*RNAi*^ transgene under control of UAS. Various RNAi lines (indicated by *UAS-X^RNAi^*) can be used to target genes in these cells to generate mosaics (Adapted from Bosch *et al.* 2015) and (ii) the possible outcomes and inferences from the generated mosaics. (D) Graphical representation of (i) whole-eye expression of Gal4 under the *Actin 5c* promoter. In these eye discs, recombination at the FRT sites (yellow triangles) results in activation of Gal4 in all cells expressing FLP under the eyeless promoter and (ii) the possible outcomes and inferences from this.

Of the many different techniques available for generation of mosaic clones, the recently described CoinFLP method offers the advantage of using RNAi lines under Gal4/UAS control (Bosch *et al.* 2015). In brief, FLPase expressed under the *eyeless* promoter can facilitate recombination through two different FRT sites (the same FLPase can enable either FRT-FRT or FRT3-FRT3 recombination) in a stochastic manner. This leads to the generation of a reliable ratio of (a) wild-type cells wherein the stop cassette is retained between the *Actin 5c* promoter and the downstream Gal4 sequence, resulting in lack of Gal4 transcription and (b) cells that have lost the stop cassette and therefore express Gal4 under the *Actin 5c* promoter (Figure 2C(i)). The use of the *eyeless* promoter to control the expression of FLPase ensures that the Gal4 is also expressed in a developmentally controlled manner in the eye imaginal discs. Cells expressing Gal4 can be marked in adult *Drosophila* eyes by the knockdown of the *white* gene using the *UAS-white^RNAi^* transgene. RNAi lines against various phosphoinositide metabolizing enzymes were tested in this background.

In order to obtain flies of the desired genotypes, virgins of *eyFLPase*, *UAS-dcr2*; *Sp/CyO*; *UAS-white^RNAi^* flies were crossed to *CoinFLP-Gal4*, *UAS-GFP* males and progeny with the genotype *eyFLPase*, *UAS-dcr2*; *CoinFLP-Gal4*, *UAS-GFP/CyO*; *UAS-white^RNAi^* were collected. Henceforth, this genotype will be referred to as the CoinFLP tester line. Males of the CoinFLP tester line were crossed to virgins of various RNAi lines, whose progeny were then screened. For controls, males of the CoinFLP tester line were crossed to wild-type (*ROR*) virgins. Female progeny without the *CyO* balancer were collected and imaged.

### Knockdown of genes in the whole eye

We used parent fly stocks containing *eyFLPase*, *UAS-dcr2*; *Sp/CyO*; *UAS-white^RNAi^* and *Act>y^+^>Gal4*, *UAS-GFP*. By employing common fly genetic schemes, we generated an *eyFLPase*, *UAS-dcr2*; *Act>y^+^>Gal4*, *UAS-GFP/CyO* line. Males from this line were crossed to either wild-type (*ROR*) virgins or selected RNAi lines. Female progeny without the *CyO* balancer were collected and imaged for eye size measurements.

### Imaging and data analysis

Flies were cold-anesthetized, their heads cut using a scalpel and then affixed to a glass slide using colorless nail varnish. Brightfield and fluorescence images were acquired using an Olympus SZX12 stereomicroscope and a 0.9X objective (effective magnification of 63X) connected to a QIClick CCD camera (QImaging, Canada) controlled via MicroManager. ImageJ software was used to measure the size of the eyes where indicated and Graphpad Prism was used to plot the graphs.

### Data availability

The authors affirm that all data necessary for confirming the conclusions of this article are represented fully within the article and its tables and figures.

## Results and Discussion

### Strategy of the genetic RNAi screen

In order to identify novel regulation of cell growth by phosphoinositide signaling, we first identified 71 RNAi lines from the Vienna Drosophila Resource Center (VDRC) collection (Dietzl *et al.* 2007), comprising both GD and KK lines, that corresponded to 32 genes of the phosphoinositide signaling toolkit (Figure 2A). These RNAi lines were crossed to the CoinFLP tester line containing *ey-FLP*, *UAS-dcr2*, *UAS-GFP*, *UAS-white^RNAi^* and *CoinFLP-Gal4*. The CoinFLP system generates roughly proportional patches of knockdown clones and otherwise wild-type clones within the developing eye tissue. Any gene that has a role in regulating cell growth or fitness would be expected to show a deviation in the ratio of the size of knockdown clones to that of wild-type clones (Figure 2C(ii)). We imaged the progeny from the crosses as described and qualitatively assessed the relative representation of knockdown clones [marked by the presence of both white (*white^RNAi^* expressing) and fluorescent (GFP expressing) ommatidia] in the adult eye. It was observed that the relative representation of knockdown clones showed a deviation in 32 RNAi lines targeting 21 genes when compared to control eyes, which had roughly 50% white/fluorescent ommatidia. Following this, we further targeted these 21 genes using a second set of RNAi lines from the Bloomington TRiP collection (Perkins *et al.* 2015). A similar analysis of the relative representation of the knockdown and wild-type clones in the eye tissue resulted in a final shortlist of 11 candidate genes that may have a role in regulating cell growth (Figure 2B). The results of both the initial screen using VDRC lines and the subset of genes screened using TRiP lines have been summarized in Table 1.

The phenotypes observed in this screen could be a consequence of either perturbations in cell intrinsic pathways that regulate growth or alterations in pathways affecting cell-cell interactions. Smaller or larger knockdown clones in the adult eye could result either from an increase or decrease in the size and/or division of cells that underwent gene knockdown. Alternatively, such a scenario could also be expected if, during development, the knockdown cells had a competitive growth advantage or disadvantage when compared to the wild-type cells within the same tissue. We employed a second screening assay to distinguish among these possibilities for the identified candidate genes. Upon whole-eye knockdown, we expect that genes that have a role in cell competition will result in normal eyes, comparable to the wild-type control flies. On the other hand, genes that are important for growth in a cell autonomous manner are expected to form smaller or larger eyes upon whole-eye knockdown (Figure 2D(ii)). We performed an RNAi screen for the 11 candidate genes using an *eyFLPase* strain that activates Gal4 expression from *Act>y^+^>Gal4* uniformly in the entire developing eye tissue (Figure 2D(i)). For each gene, the RNAi line that showed the strongest phenotype in the mosaic screen was chosen for this assay. The eyes of the female progeny were imaged and the size of the eyes in control and knockdown flies was determined.

### Phosphoinositide-metabolizing genes regulating cell growth

Of the 11 genes identified from the CoinFLP screen, three genes – *PI4KIIIα* (PI4 kinase), *Sac1* (PI4P phosphatase) and *Pis* (PI synthase) – are known to be important for cell survival. Disruption of *PI4KIIIα* results in embryonic lethality in both flies (Tan *et al.* 2014) and mice (Nakatsu *et al.* 2012). *PI4KIIIα* mutant clones in the eye discs show cell death (Yan *et al.* 2011). Eyes also fail to develop in *PI4KIIIα* null whole-eye mosaics, suggesting that complete loss of PI4KIIIα function leads to cell lethality (Liu *et al.* 2018; Balakrishnan *et al.* 2018). *Sac1* mutant *Drosophila* are embryonic lethal (Wei *et al.* 2003b) due to defects in dorsal closure (Wei *et al.* 2003a). Growing temperature-sensitive mutant flies of *Sac1* at restrictive temperatures resulted in death of adult flies within one to three days post eclosion (Del Bel *et al.* 2018). *Sac1* mutant clones generated in larval wing discs show activation of Caspase 3 as a result of active JNK signaling (Yavari *et al.* 2010) and downregulation of *Sac1* in the nervous system leads to pupal lethality (Forrest *et al.* 2013). The observation that knockdown of both PI4KIIIα, which converts PI to PI4P, and Sac1, which performs the reverse reaction of converting PI4P to PI, lead to cell death suggests that the levels of PI4P are under strict regulation and changes in these levels through loss of either enzymatic activity affects cell survival. Disruption of *Pis*, the key enzyme that catalyzes conversion of cytidine diphosphate diacylglycerol (CDP-DAG) to phosphatidylinositol (PI), the precursor to all other phosphoinositides, leads to lethality in yeast (Nikawa *et al.* 1987) and embryonic lethality in flies (Wang and Montell 2006). Generation of *Pis* mutant eyes in an otherwise heterozygous fly resulted in smaller eyes with a rough eye morphology, suggesting that loss of *Pis* also leads to cell lethality (Wang and Montell 2006). In accordance with these previous studies, we found that whole-eye knockdown of these genes leads to pupal lethality.

**Figure 3:**
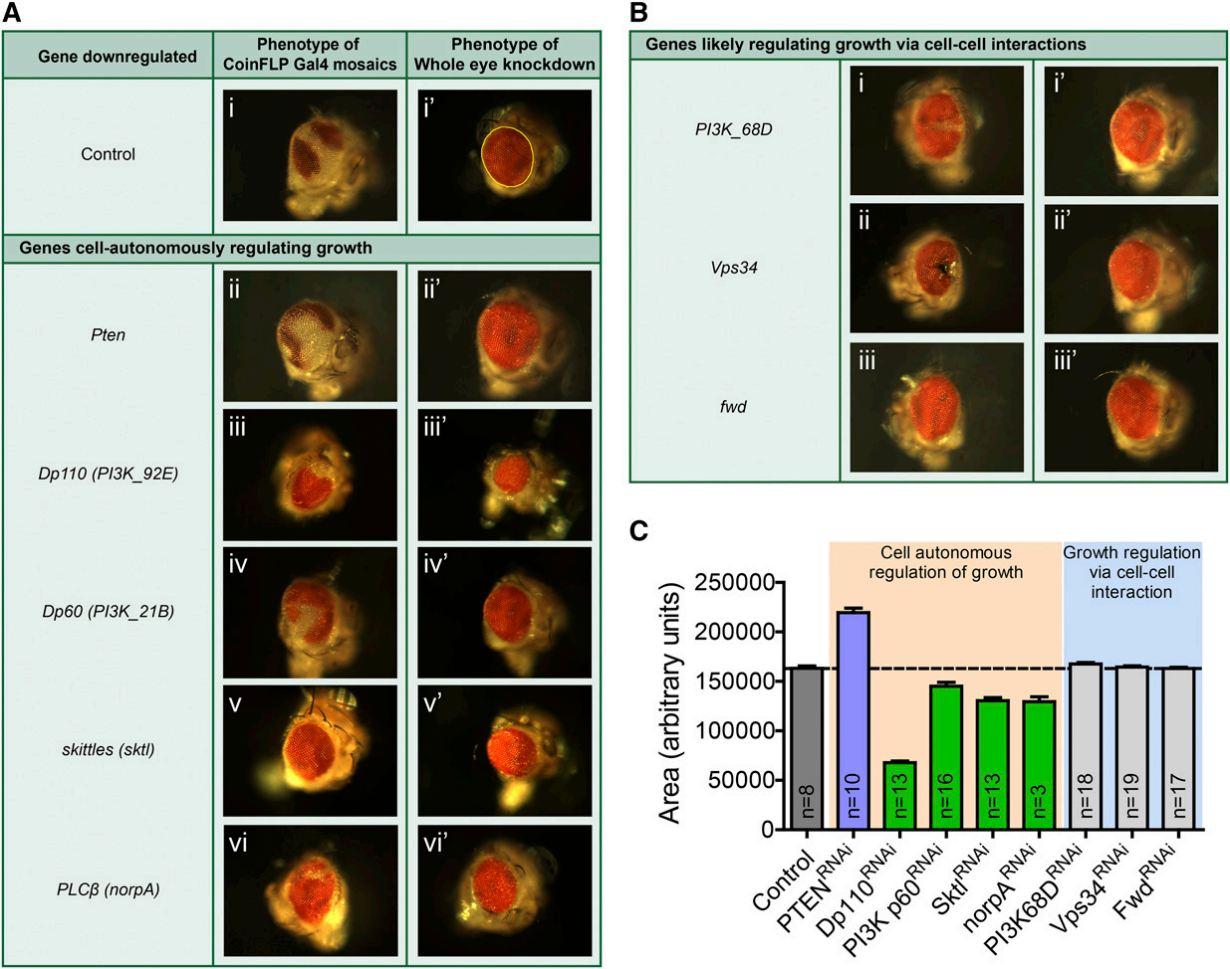
Hits identified from the screen. Representative images of (A) controls and genes that were identified to cell autonomously regulate growth. (B) Genes identified to regulate cell growth via cell-cell interaction. (C) Area of eyes after whole-eye knockdown of genes was determined by drawing an outline as indicated in Figure 3A(i’) and plotted. Dotted line indicates the area of control eyes for comparison with those of tested RNAi lines.

We classified the remaining hits on the basis of the phenotypes observed in the primary mosaic screen and the secondary whole-eye knockdown assay as genes that (A) have a cell-autonomous/intrinsic effect on cell growth (B) that possibly regulate growth through cell-cell interactions (Figure 3).

### Genes that cell-autonomously regulate growth

Of all the genes tested in the mosaic screen, interestingly, only knockdown of *Pten* led to an increase in the representation of knockdown cells as compared to wild-type cells in the eye tissue (Figure 3A(ii)). Knocking down *Pten* in whole eyes resulted in larger eyes when compared to control flies (Figures 3A(ii’) and 3C). It has already been demonstrated that in mitotic tissues of *Drosophila*, homozygous mutant clones of *Pten* have large cells. Moreover, the clones seen in those studies were larger due to an increase in cell number (Huang *et al.* 1999; Goberdhan *et al.* 1999). We observed similar phenotypes upon knockdown of *Pten* in the mosaic screen where not only did knockdown cells form a larger fraction of adult eye tissue, but the individual *Pten* knockdown ommatidia also seemed larger than wild-type control cells. Thus, as a proof of principle, the results from our screen validate the cell autonomous role of PTEN as a negative regulator of cell growth and proliferation.

Apart from PTEN, we observed the catalytic subunit of Class I PI3K (*Dp110*), the regulatory subunit of Class I PI3K (*Dp60*), PI4P5K – CG9985 (*skittles*) and PLCβ (*norpA*) to have cell autonomous effects on cell growth. Of these, only the Class I PI3K subunits have been previously demonstrated to have such effects. The subunits form a heterodimeric complex in cells where the Dp110 is the catalytic subunit and Dp60 acts as the regulatory subunit. Upon activation by upstream signals like receptor tyrosine kinases, Class I PI3K utilizes PI(4,5)P_2_ to form PI(3,4,5)P_3_, which can in turn activate downstream effectors that regulate growth-related processes. Loss of either *Dp110* or *Dp60* results in reduced size of cells, whereas overexpression of *Dp110* results in an autonomous increase in size and number of cells. Interestingly, overexpression of the *Dp60* subunit results in a decrease in the size and proliferation of cells through a mechanism that is still not clearly understood (Weinkove *et al.* 1999). Overexpression of a catalytically dead form of *Dp110* produces a dominant-negative effect by reducing the size and number of cells, whereas a plasma-membrane targeted form of Dp110 is even more effective than the wildtype Dp110 in driving cell growth and proliferation (Leevers *et al.* 1996). The results from our study corroborate these observations. Cells that were depleted of Dp110 (Figure 3A(iii)) or Dp60 (Figure 3A(iv)) produced very small clones in the CoinFLP mosaic screen. Moreover, depleting Dp110 (Figures 3A(iii’) and 3C) and Dp60 (Figures 3A(iv’) and 3C) in the whole eye resulted in smaller eyes, with Dp110 manipulations resulting in more severe phenotypes in each case.

Loss of s*kittles* (*sktl*) in the CoinFLP screen led either to the presence of very small clones or to a complete loss of knockdown clones in the eye (Figure 3A(v)), whereas the whole-eye knockdown of *sktl* resulted in smaller eyes (Figures 3A(v’) and 3C). SKTL is the *Drosophila* ortholog of PI4P5K that converts PI4P to PI(4,5)P_2_. Mutant alleles of *sktl* are either embryonic or larval lethal (Hassan *et al.* 1998). Using transheterozygotic mutant allele combinations, including the most severe but viable alleles, studies have demonstrated that *sktl* is dispensable for nervous system development, neurotransmitter release and normal electrical response to light in *Drosophila* photoreceptors (Hassan *et al.* 1998; Chakrabarti *et al.* 2015). Observations from our screen using sktl^*RNAi*^ lines suggest that *sktl* is required for cell viability or proliferation during eye disc development. This is in agreement with previous studies that report a failure to obtain *sktl* mutant clones in eye and wing imaginal discs (Hassan *et al.* 1998). However, *sktl* was identified as an apoptotic effector in a screen performed in *Drosophila* S2R+ cells, where *sktl* knockdown showed a mild but statistically significant inhibitory effect on apoptosis (Chew *et al.* 2009). In *Drosophila* ovarian follicular cells, SKTL appears to play an important role in regulating the localization of PAR-3, a member of the master polarity regulator complex, by maintaining PI(4,5)P_2_ levels and thus defining the apico-lateral boundary. Reduction in PI(4,5)P_2_ levels upon loss of *sktl* alters PAR-3 localization and decreases the size of the apical domain, eventually leading to delamination and loss of s*ktl* mutant clones. However, no difference in proliferation or apoptosis was observed in these clones (Claret *et al.* 2014). Therefore, further experiments would be necessary to investigate the mechanisms leading to loss of *sktl* knockdown clones in the developing eye.

*norpA* (*PLCβ*) came up as an unexpected hit in our screen for regulators of growth (Figure 3A(vi)). PLCs hydrolyze PI(4,5)P_2_ to generate second messengers Diacylglycerol (DAG) and Inositol 1, 4, 5 trisphosphate (IP_3_). Antisense RNA-mediated suppression of mammalian PLC isoforms β, δ and γ has been reported to result in increased PI(4,5)P_2_ levels and inhibition of cell growth (Nebigil 1997). In flies however, *norpA* mutants are reported to have normal sized eyes and have been used extensively to study phototransduction (Yoshioka *et al.* 1985). In contrast to this, whole-eye knockdown of *norpA* resulted in smaller eyes (Figures 3A(vi’) and 3C). This prompted us to take a closer look at the norpA^*RNAi*^ lines used in our study.

The VDRC norpA^*RNAi*^ line that gave the strongest phenotype (VDRC 21490) in the CoinFLP screen (and hence, was used for the whole-eye knockdown) is no longer available with VDRC. The other VDRC norpA^*RNAi*^ line (VDRC 105676) has a predicted off-target effect on the gene *frazzled* (CG8581), important for axon and dendritic guidance. The two TRiP norpA^*RNAi*^ lines had either no effect or very mild effects in the CoinFLP screen. We therefore conclude that *norpA* is not a real hit and is most likely an artifact of off-target effects of some RNAi lines, thus highlighting the strength of the use of multiple RNAi lines against each gene in our screen.

### Genes that likely regulate growth via cell-cell interactions

As part of the two-step screen we identified a small set of genes where the RNAi-mediated knockdown clones for these genes were smaller than the wild-type clones in the mosaic CoinFLP screen. However, whole-eye knockdown of the same set of genes failed to show any effect upon the adult eyes, which remained similar in size when compared to control flies. This indicated that such genes might support cell growth and/or survival through cell-cell signaling, including mechanisms that involve cell-cell competition. *Pi3K68D*, *Vps34* and one of the PI4Ks – *four wheel drive* (*fwd*) – fell in this category.

*Pi3K68D* codes for a Class II PI3K enzyme that has been shown to localize to the plasma membrane and endo-lysosomal structures. It utilizes PI or PI4P as substrates to synthesize PI3P or PI(3,4)P_2_, respectively (MacDougall *et al.* 1995; Velichkova *et al.* 2010). *Pi3K68D* has been previously shown to regulate patterning in *Drosophila* wing imaginal discs but did not affect eye imaginal discs under the conditions tested. Genetic interactions of PI3K68D with EGF receptor and Notch signaling pathways were seen to be important for this regulation of patterning (MacDougall *et al.* 2004). No study directly links Class II PI3K to cell growth or survival in *Drosophila*. In HeLa cells and CHO cells, downregulation of PI3K-C2a, one of the three mammalian Class II PI3K isoforms, results in increased apoptosis (Kang *et al.* 2005; Elis *et al.* 2008). However, contrary to this, downregulation of PI3K-C2a in human muscle cells, human lung epithelial fibroblasts and rat insulinoma cells shows no effect on proliferation (Elis *et al.* 2008; Dominguez *et al.* 2011). While our initial mosaic screen suggested that loss of *PI3K68D* (Figure 3B(i)) may lead to apoptosis as seen in HeLa or CHO cells, this was unlikely as knocking down *PI3K68D* had no effect in whole eyes (Figures 3B(i’) and 3C). Our screen therefore implicates *PI3K68D* as an important regulator of cell-cell interaction and the underlying mechanism, if investigated, may reveal novel modes of growth regulation.

Vps34 is a Class III PI3K that converts PI to PI3P on endosomes. In mammalian cells, signaling via Vps34 is important for the transduction of amino acid and glucose signals into mTORC1 output (Byfield *et al.* 2005; Nobukuni *et al.* 2005) which further regulates cell growth. In such a scenario, Vps34 would be expected to autonomously regulate cell growth via mTORC1 signaling. In *Drosophila*, while the requirement of mTOR activity to mediate amino acid sensing into growth is conserved (Zhang *et al.* 2000), Vps34 has been reported to be dispensable for normal mTOR signaling in fat body cells.

Vps34 also plays an important role in the regulation of autophagy (Juhász *et al.* 2008). Autophagy is shown to be both pro-survival and pro-death in a context dependent manner (Denton *et al.* 2011). Reduction of autophagy reduces cell death in larval salivary glands (Denton *et al.* 2013). Similarly, knockdown of many genes involved in autophagy, including *Vps34*, delays the programmed cell death of obsolete *Drosophila* larval midgut (Xu *et al.* 2015). In contrast to these, we saw that the mosaic clones of *Vps34* were smaller than controls (Figure 3B(ii)) suggesting that Vps34 has a pro-survival role in the developing eye tissue. It is likely that an interplay between mTORC1-dependent regulation of cell growth and mTOR-independent regulation of autophagy decides the fate of *Vps34* knockdown cells.

In addition, our results suggest that *Vps34* has a role in cell competition as whole-eye knockdown of *Vps34* did not result in a reduction in the size of the eye (Figures 3B(ii’) and 3C)despite an under representation of clones in the CoinFLP screen. Epithelial cells with disrupted apicobasal polarity are known to be eliminated by neighboring wild-type cells by the process of cell competition (Di Gregorio *et al.* 2016) during which JNK activation is seen in ‘loser cells’ (Amoyel and Bach 2014). Loss of Vps34 results in activation of JNK pathway, leading to disruption of epithelial organization (O’Farrell *et al.* 2017). Taken together, these studies hint toward the possibility that Vps34 knockdown leads to JNK activation mediated disruption of apicobasal polarity and loss of cells.

The *Drosophila* genome harbors one gene each for the three families of PI4 kinases (PI4Ks). The three families of PI4 kinases produce PI4P using PI as a substrate at distinct intracellular membranes. Among the three genes, *viz*. *fwd*, *Pi4KIIα* and *PI4KIIIα*, we observed phenotypes only upon knockdown of *fwd* and *PI4KIIIα*. As mentioned earlier, loss of *PI4KIIIα* resulted in a complete loss of knockdown clones in the mosaic screen and led to pupal lethality when it was downregulated in the entire eye tissue. As a result, it appears likely that PI4KIIIα is essential for cellular viability. On the other hand, smaller knockdown clones were observed when *fwd* was downregulated in the mosaic CoinFLP Gal4 screen (Figure 3B(iii)). However, like *PI3K68D* and *Vps34*, downregulation of *fwd* across the entire developing eye failed to show any significant phenotypes (Figures 3B(iii’) and 3C), again suggesting cell-cell interactions between *fwd*-deficient and neighboring wild-type cells to be the likely reason for reduced size of fwd^*RNAi*^ clones. *fwd* knockout flies are viable and female fertile. *fwd* knockout male flies are sterile due to defects in cytokinesis during male meiosis (Brill *et al.* 2000). Both fly and mammalian *fwd* (PI4Kβ) bind and recruit Rab11 to the Golgi and are required for the maintenance of Golgi integrity and secretion (de Graaf *et al.* 2004; Giansanti *et al.* 2007; Polevoy *et al.* 2009). These reports suggest that *fwd* may have pleiotropic cellular roles, causing the phenotypes to vary depending on the tissues in which its levels are manipulated.

In summary, our screen identified several components of the phosphoinositide metabolism toolkit as regulators of cell growth. Using the power of mosaic analysis in the *Drosophila* eye, we were able to classify these into those exerting their effect in a cell-autonomous manner and those likely acting via cell-cell interactions in a plane of developing cells. Our screen identified three genes that may regulate growth via cell-cell interactions. These include *Pi3K68D*, *Vps34* and *fwd*. Interestingly, *Pi3K68D* is found only in a subset of metazoans, Bilateria. The observation that *Pi3K68D* is not present in single cell eukaryotes but are only found in multicellular eukaryotes further supports our findings that *Pi3K68D* may have a role in cell-cell interactions. The products of the three identified enzymes, PI(3,4)P_2_, PI3P and PI4P, have so far not been directly linked to cell competition. The identification of these genes as regulators of growth has thus opened up new links between phosphoinositide metabolizing enzymes and cell growth that invites further studies to explore underlying mechanisms.

The current screen included phosphoinositide kinases, phosphatases and a few other phosphoinositide metabolizing enzymes. However, signaling events downstream of their generation are dependent on the ability of these lipids to bind target proteins and modulate their activities. There are about 70 phosphoinositide binding proteins annotated in *Drosophila* (Balakrishnan *et al.* 2015). Extending the CoinFLP screen to these phosphoinositide binding proteins in the future would further our understanding of the mechanisms by which phosphoinositides regulate growth.
